# The nature and frequency of abdominal symptoms in cancer patients and their associations with time to help-seeking: evidence from a national audit of cancer diagnosis

**DOI:** 10.1093/pubmed/fdx188

**Published:** 2018-01-27

**Authors:** Minjoung Monica Koo, Christian von Wagner, Gary A Abel, Sean McPhail, William Hamilton, Greg P Rubin, Georgios Lyratzopoulos

**Affiliations:** 1 University College London, Torrington Place, London, UK; 2 University of Exeter Medical School, St Luke’s Campus, Heavitree Road, Exeter, UK; 3 National Cancer Registration and Analysis Service, Public Health England Zone A, 2nd Floor, Skipton House, 80 London Road, London, UK; 4 Institute of Health and Society, Newcastle University, Sir James Spence Institute, Royal Victoria Infirmary, Newcastle upon Tyne, UK

**Keywords:** cancer, health promotion, public health

## Abstract

**Background:**

Raising awareness of possible cancer symptoms is important for timely help-seeking; recent campaigns have focused on symptom groups (such as abdominal symptoms) rather than individual alarm symptoms associated with particular cancer sites. The evidence base supporting such initiatives is still emerging however; understanding the frequency and nature of presenting abdominal symptoms among cancer patients could inform the design and evaluation of public health awareness campaigns.

**Methods:**

We examined eight presenting abdominal symptoms (abdominal pain, change in bowel habit, bloating/distension, dyspepsia, rectal bleeding, dysphagia, reflux and nausea/vomiting) among 15 956 patients subsequently diagnosed with cancer in England. We investigated the cancer site case-mix and variation in the patient interval (symptom-onset-to-presentation) by abdominal symptom.

**Results:**

Almost a quarter (23%) of cancer patients presented with abdominal symptoms before being diagnosed with one of 27 common and rarer cancers. The patient interval varied substantially by abdominal symptom: median (IQR) intervals ranged from 7 (0–28) days for abdominal pain to 30 (4–73) days for dysphagia. This variation persisted after adjusting for age, sex and ethnicity (*P* < 0.001).

**Conclusions:**

Abdominal symptoms are common at presentation among cancer patients, while time to presentation varies by symptom. The need for awareness campaigns may be greater for symptoms associated with longer intervals to help-seeking.

## Introduction

Diagnosing cancer early in symptomatic patients is a prominent feature of contemporary cancer control strategies.^1,2^ A range of pioneering studies during the last decade have established associations between the knowledge (‘awareness’) of likely symptoms of cancer among the general public and timely presentation, diagnosis, and outcomes.^3–6^ Public health agencies have consequently implemented educational interventions aimed at raising awareness of cancer symptoms in order to promote timely presentation.^7–9^ However, the evidence base supporting the design of such interventions is still emerging.

Previous symptom awareness campaigns have tended to take a cancer-based approach, by targeting ‘red-flag’ or ‘alarm’ symptoms explicitly associated with specific cancers, such as ‘blood in poo’ and colorectal cancer.^10–12^ There is however growing interest in targeting symptoms relating to a body area or system, partly as this provides an opportunity to promote the earlier presentation of rarer and less common cancers. In England, an abdominal symptoms campaign was recently piloted at regional level, focusing on a range of symptoms (diarrhoea, bloating, abdominal discomfort, constipation, nausea, and blood in poo).^13^

Examining the length of the patient interval (time from symptom onset to presentation) associated with different abdominal symptoms can contribute to the design of future campaigns. Awareness campaigns about possible cancer symptoms aim to shorten the patient interval by encouraging timely symptom appraisal and help-seeking.^14^ Therefore, symptom-specific patient intervals may be interpreted as measures of relative need for such interventions.^15^ Alongside considerations of other important factors such as the predictive value of a symptom for cancer, and the prevalence of different symptoms in the general population, such evidence can support how the content of awareness campaigns could prioritize certain symptoms over others.

Further, estimating the impact of a symptom awareness campaign has been shown to be challenging due to the diffuse and broad-reaching nature of campaigns; such difficulties are likely to be exacerbated by symptom-based approaches that target more than one cancer site.^7,16^ Evidence regarding the anticipated cancer site case-mix of a particular symptom could help guide the direction of evaluation strategies, though such evidence is generally lacking.^17^

We therefore examined the frequency of abdominal symptoms at presentation in a representative population of incident cancer patients; described the range of cancers associated with abdominal symptoms in an incident cohort; and examined variation in the length of the patient interval by presenting abdominal symptom.

## Methods

### Data source

We used data from the first English cancer audit (National Audit of Cancer Diagnosis in Primary Care) 2009–10, details of which have been described previously.^18^ Briefly, participating clinicians collected information on the diagnostic process for incident cancer patients in ~14% of all general practices in England, excluding screen-detected cases. The audited cancer patient population was representative of incident cancer patients in England during the same period, while the characteristics of participating practices were found to be comparable to non-participating practices.^18,19^

### Patient population

We analysed data from cancer patients with complete and valid information on age group (among patients aged 15 years or older), sex, presenting symptoms and cancer site (see Supplementary Fig. S1 for flow chart of sample derivation). Individuals diagnosed incidentally and those with cancer sites categorized as ‘No information’ and ‘Unknown Primary’ were excluded from the analysis. Among the 3661 cancer patients with one or more abdominal symptoms, 2936 (80%) had complete information on the patient interval (see Supplementary Table S2 for the proportion of missing values by individual symptom). Overall, the strongest predictor of missing interval or pre-referral consultation data was first presentation to a healthcare facility other than the patient’s own general practice, without evidence for substantive differences by socio-demographic characteristic (data not shown).

### Variables of interest

General practitioners participating in the audit provided free text answers to the question ‘what was the main presenting symptom?’ for each patient, based on information in their primary care records. As described previously,^20^ we coded symptom constructs following principles of natural language processing (NLP), without prior definitions or restrictions regarding cancer-symptom associations.^21^ If multiple symptoms were mentioned, they were assumed to be synchronous. Symptoms were initially assigned by MMK, and cross-validated by GL and GPR, an approach also used previously.^20^ Based on the abdominal symptoms described by the 2015 National Institute for Health and Care Excellence (NICE) guidelines for suspected cancer, we selected a total of 18 symptom constructs (see Supplementary Table S2) which were further aggregated into eight abdominal symptom groups: (non-acute) abdominal pain, bloating or distension, change in bowel habit, dysphagia, dyspepsia, nausea or vomiting, rectal bleeding and reflux.^22^

The patient interval was defined as the number of days between symptom onset and the first presentation to primary care, in line with the Aarhus Statement.^23^

### Statistical analysis

The frequency (and associated exact confidence intervals) of abdominal symptoms in the studied population of cancer patients were estimated. We then described the cancer site case-mix of abdominal symptoms, namely the range and relative frequencies (proportions) of different cancer sites subsequently diagnosed among cancer patients presenting with abdominal symptoms.

Subsequently, we examined variation in the patient interval by abdominal symptom. As public awareness campaigns target individual symptoms rather than symptom combinations, these analyses were restricted to the majority of cancer patients with a single recorded presenting abdominal symptom (*n* = 2253, 62% of all patients reporting an abdominal symptom)—though we examined common abdominal symptom combinations in supplementary analyses. Firstly, the mean, median, interquartile range and 90th centiles of the patient interval were estimated for each abdominal symptom along with 95% confidence intervals using a bootstrap approach with 1000 replications. Kruskal–Wallis tests were used to test variation in median interval length by abdominal symptom. The proportion of patients with each symptom that experienced a patient interval of 60 days or longer was also calculated to help to further contextualize the findings.

We then used generalized linear models (GLM) to examine the association between abdominal symptoms and the patient interval adjusted for age group (parameterized as <50 years, 50–69 years, 70+ years), ethnicity (white, non-white) and sex (men, women) given prior evidence supporting their associations with diagnostic timeliness.^24^ To account for skewed outcome data, a log link function was used (which allows the covariates to be modelled on a linear additive scale, aiding interpretation), and significance testing was based on bootstrapping (1000 replications). Variation in interval length was examined using joint Wald tests, with statistical significance at the 5% level. All analyses were conducted using STATA SE v 13.1 (StataCorp, College Station, TX, USA).

### Supplementary analyses

We conducted supplementary analyses examining the frequency of 12 most common abdominal symptom combinations, and their associated distributions of the observed patient interval in the same way as described above.

## Results

### Frequency of presenting abdominal symptoms in cancer patients

Of a total of 15 956 patients with cancer, 3661 (23%) presented with one or more abdominal symptoms. Abdominal pain was the most common abdominal symptom across the entire cohort of cancer patients (8%), followed by change in bowel habit (6%), and rectal bleeding (5%) (Table 1).

**Table 1:** Frequency of abdominal symptoms among symptomatic cancer patients (*n* = 15 956)

Symptom	No. of patients	Percentage of symptomatic cancer patients (95% CI)
Abdominal pain	1268	7.9 (7.5–8.4)
Change in bowel habit	1010	6.3 (6.0–6.7)
Rectal bleeding	768	4.8 (4.5–5.2)
Dysphagia	418	2.6 (2.4–2.9)
Nausea or vomiting	261	1.6 (1.5–1.8)
Dyspepsia	256	1.6 (1.4–1.8)
Bloating or distension	250	1.6 (1.4–1.8)
Reflux	71	0.4 (0.4–0.6)
Any abdominal symptom	3661	22.9 (22.3–23.6)

NB the number of patients (percentages) sum to more than 3661 (23%) as patients could have more than one abdominal symptom.

### Cancer site case-mix of abdominal symptoms in cancer patients

**Fig. 1:**
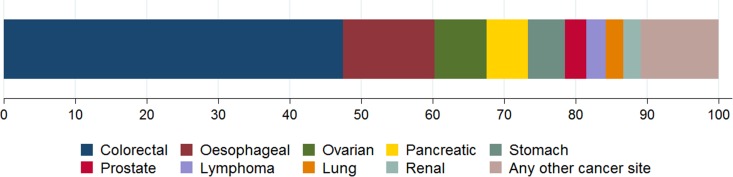
Cancer site case-mix of patients who presented with one or more abdominal symptom (*n* = 3661). NB Proportions of the nine most frequent cancers across all abdominal symptoms shown only; other cancer diagnoses are represented as ‘Any other cancer site’ category. See Table 2 for exact proportions.

Among the 3661 cancer patients who presented with abdominal symptoms, the majority (89%, 3244/3661) were diagnosed with solid cancers of abdominal or adjacent organs (Fig. 1). The most commonly diagnosed cancer site was colorectal cancer (47%), followed by oesophageal (13%), ovarian (7%) and pancreatic (6%) cancers (Table 2 and Fig. 1). A further 14 cancer sites were represented among the remainder of patients, including solid tumours of non-abdominal (and non-adjacent) organs (8%) and haematological cancers (4%).

**Table 2:** Cancer site case-mix of patients with one or more abdominal symptoms (n=3661) and proportion of patients with a given cancer that had abdominal symptoms

Cancer	Number of patients	Percentage of patients with one or more abdominal symptoms subsequently diagnosed with a given cancer (95% CI)	Percentage of patients with a given cancer who had one or more abdominal symptoms
Abdominal cancers^a^			
Colorectal	1737	47.4 (45.8–49.1)	75 (73–77)
Oesophageal	468	12.8 (11.7–13.9)	84 (80–87)
Ovarian	267	7.3 (6.5–8.2)	70 (65–74)
Pancreatic	214	5.8 (5.1–6.7)	59 (54–64)
Stomach	189	5.2 (4.5–5.9)	65 (60–71)
Prostate	110	3.0 (2.5–3.6)	5 (4–6)
Renal	89	2.4 (2.0–3.0)	29 (24–34
Bladder	40	1.1 (0.8–1.5)	5 (4–7)
Liver	38	1.0 (0.8–1.4)	44 (34–54)
Small intestine	36	1.0 (0.7–1.4)	69 (56–80)
Gallbladder	32	0.9 (0.6–1.2)	51 (39–63)
Endometrial	24	0.7 (0.4–1.0)	6 (4–9)
Sub-total	3244	88.6 (87.5–89.6)	41.0 (39.9–42.1)
Other cancers			
Lung	91	2.5 (2.0–3.0)	5 (4–6)
Oropharyngeal	20	0.5 (0.4–0.8)	10 (6–14)
Breast	14	0.4 (0.2–0.6)	0.5 (0.3–0.9)
Laryngeal	12	0.3 (0.2–0.6)	10 (6–17)
Brain	10	0.3 (0.1–0.5)	5 (3–8)
Cervical	10	0.3 (0.1–0.5)	8 (4–14)
Sarcoma^b^	10	0.3 (0.1–0.5)	10 (5–17)
Testicular	5	0.1 (0.1–0.3)	3 (1–8)
Melanoma	4	0.1 (0.04–0.3)	0.5 (0.2–1.3)
Mesothelioma	4	0.1 (0.04–0.3)	6 (2–14)
Thyroid	4	0.1 (0.04–0.3)	4 (2–10)
Sub-total	279^c^	7.6 (6.8–8.5)^c^	4.1 (3.6–4.6)^c^
Haematological cancers			
Lymphoma^b^	97	2.6 (2.2–3.2)	15 (12–18)
Leukaemia	25	0.7 (0.5–1.0)	7 (5–11)
Myeloma	16	0.4 (0.3–0.7)	8 (5–13)
Sub-total	138	3.8 (3.2–4.4)	11.5 (9.8–13.4)
Total	3661^c^	100^c^	23^c^

^a^Defined as cancers arising in the intra-abdominal organs, together with oesophageal and prostate cancer NB ordered by frequency among patients with abdominal symptoms.

^b^It is likely that a proportion of sarcomas and lymphomas were intra-abdominal but information regarding their exact location was not available.

^c^Includes 95 cases described as ‘Other’ cancers.

We also considered the relative importance of abdominal symptoms for each cancer site by calculating the proportion of patients with a given cancer who had presented with one or more abdominal symptoms. Unsurprisingly, over two-fifths (41%) of cancer patients diagnosed with an abdominal cancer had presented with abdominal symptoms, although this ranged from 84% of patients later diagnosed with oesophageal cancer to 5% of patients later diagnosed with prostate cancer (see Table 2 for full breakdown). Patients with cancers arising outside the abdominal region were much less likely to report abdominal symptoms (4%, *n* = 279). In contrast, patients diagnosed with haematological cancers were relatively more likely to report abdominal symptoms at presentation (11%, *n* = 138), almost two-thirds of those being patients with lymphoma (Table 2).

### Patient interval by presenting abdominal symptom

**Fig. 2:**
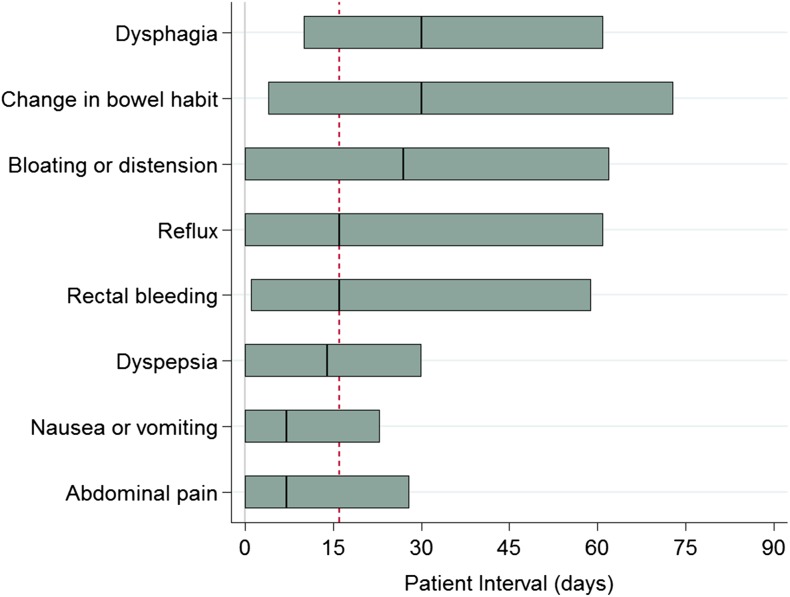
The length of the patient interval by presenting abdominal symptom (ordered by median interval; bar length = IQR, vertical line = median value). The dashed vertical line represents the median interval value across all patients with abdominal symptoms (16 days). For corresponding values please see Supplementary Table S3 in the Supporting information.

Among cancer patients with a single presenting abdominal symptom (*n* = 2253), there was strong evidence for variation in the patient interval (symptom-onset-to-presentation) by symptom (*P* < 0.001, Fig. 2 and Supplementary Table S3). Patients presenting with change in bowel habit or dysphagia had the longest patient intervals: one in two patients with either of these symptoms waited at least a month before presentation, while a quarter waited 2 months or longer (median (IQR) patient interval: 30(4–73) days for change in bowel habit; and 30(10–61) days for dysphagia). A considerable proportion (25–30%) of patients with bloating or distension, reflux and rectal bleeding also waited for two months or longer before presentation. In contrast, cancer patients presenting with abdominal pain or nausea/vomiting went to the doctor sooner on average (7(0–28) days and 7(0–23) days, respectively). The variation in interval length by abdominal symptom persisted after adjusting for age group, sex and ethnicity (Supplementary Table S4).

In supplementary analyses, we considered the 12 most common categories of single or combinations of presenting symptoms, including 3438 patients (94% of patients reporting one or more abdominal symptom). Results were largely comparable to the main analyses finding in respect of associations with the patient interval (see Supplementary Tables S5 and S6).

## Discussion

### Main findings of this study

Almost one in four cancer patients presented with abdominal symptoms before diagnosis. The majority of cancer patients who presented with abdominal symptoms were subsequently diagnosed with a range of common and rarer cancers of abdominal or adjacent organs, but a proportion of patients had tumours of other solid organ tumours, or haematological malignancies. The median patient interval ranged from 7 days for abdominal pain to 30 days for dysphagia. The observed differences in interval length by abdominal symptom remained when adjusted for age, sex and ethnicity.

### What is already known on this topic

In our study, colorectal, oesophageal, ovarian and pancreatic cancers accounted for the majority of cancer patients that presented with one or more abdominal symptoms, consistent with previous evidence.^25,26^ However, we also found large proportions of patients diagnosed with rarer cancers such as stomach (65%), small intestinal (69%) and gallbladder cancers (51%) presenting with abdominal symptoms.

Comparable evidence on the association between the patient interval and abdominal symptoms is limited to two English studies on colorectal and pancreatic cancers, respectively.^26,27^ Rectal bleeding and dyspepsia-like symptoms were associated with shorter time to presentation compared with other studied symptoms, in line with our findings regarding these symptoms.^26^

### What this study adds

In order to improve the timeliness of diagnosis among cancer patients who present with symptoms, we need a better appreciation of the nature and frequency of presenting symptoms among these patients; currently, related epidemiological evidence is limited in quantity and breadth^17^. Consequently, our study adds substantially to the present evidence base, both regarding the burden of abdominal symptoms in incident cancer patients, and their associations with time to help-seeking. Abdominal symptoms appear to be common among incident cases of cancer, suggesting that symptom awareness campaigns focusing on abdominal symptoms could potentially expedite the diagnosis of a large range of both common and rarer cancers.

Previous analyses have shown large variation in the patient interval by cancer site.^28,29^ Our findings suggest that this chiefly reflects variation in interval length of the most frequent symptoms of the different cancers. After considering symptom prevalence and predictive values of each symptom, variation in the length of the patient interval associated with different symptoms could help to identify particular symptoms for prioritization in campaigns. For example, we found that one in two cancer patients with dysphagia waited almost a month before presenting. As dysphagia is also an established ‘alarm’ symptom for cancer, this finding argues for its further targeting by future campaigns.^30^ In contrast, cancer patients with abdominal pain presented after a median interval of 7 days, and given its high prevalence and low predictive value, there may be little to be gained by raising its awareness amongst the general population.^31,32^

Previous evaluations have examined the increase in number of ‘2-week wait’ referrals, the corresponding conversion rates to cancer cases, and diagnostic activity.^33^ For campaigns targeting groups of symptoms, understanding the anticipated range of affected cancer sites will be crucial for accurate assessment of the campaign’s impact.

### Limitations of this study

The study design enabled analysis of data on both the presenting symptoms and associated patient intervals among a large and representative cancer patient population in England. Our findings of symptom prevalence across a representative cohort of patients diagnosed with 1 of 28 cancer sites substantially augment previous evidence dominated by cancer site-specific symptom studies.^25–27,30,34^

There are several limitations. Firstly, data on symptoms and the patient interval used in our study is reliant on the information on presenting symptoms and their duration being accurately and completely declared or elicited during consultation, and recorded in the patient’s record. Nonetheless such approaches enable the profiling of large patient groups without potential concern about recall or survivorship bias.^29,35^ A minority of patients with abdominal symptoms had missing outcome data regarding the patient interval, as noted in similar studies in this field.^26,27,36,37^

We restricted our analyses to eight abdominal symptoms based on those recommended for urgent referral in national clinical guidelines.^22^ This was a pragmatic decision that has face validity as symptom awareness campaigns are unlikely to include symptoms with a very low predictive value. We examined the patient interval among patients with a single abdominal symptom for ease of interpretation, again because campaign messages have thus far focused on single symptoms as opposed to synchronous symptom combinations. Further, in sensitivity analyses considering the most frequent symptom combination groups among nearly all cancer patients who presented with one or more abdominal symptom, we found concordant findings (see Supplementary Table S5 and S6).

Our analysis focuses on the significance of abdominal symptoms among patients subsequently diagnosed with cancer, and provides insight into how awareness campaigns may be evaluated. Nevertheless, it is clear that abdominal symptoms in primary care may represent other important diseases, such as inflammatory bowel disease.^26,38^ Coordinating our findings with evidence regarding the prevalence of abdominal symptoms among the general population, and the potential diagnostic experiences of patients that seek help for such symptoms beyond the cancer context may bring further insight.^39,40^ We were unable to examine variation in patient interval by comorbidity status or deprivation: symptom appraisal and therefore the length of the patient interval, may be influenced by the presence of other conditions, and lower socioeconomic groups tend to experience lowest symptom knowledge and longer time to presentation.^41–43^ However, such associations, if present, are unlikely to substantially confound the observed variation by abdominal symptom, which is the main focus of our study. Finally, while our findings provide insight into the associations between symptoms and timeliness of help-seeking before major population level campaigns were launched (in 2011), further examination of these associations between symptoms and timeliness of help-seeking in more recent cohorts will provide further insight.^33^

### Conclusions

In conclusion, almost a quarter of all patients with cancer initially present with an abdominal symptom, and their interval to presentation varies substantially by (abdominal) symptom type. The timeliness of presentation associated with individual symptoms could inform the design of campaigns, while the cancer site case-mix of a particular symptomatic presentation could be used to inform evaluation.

## Supplementary Material

Supplementary Data

## References

[1] Independent Cancer Taskforce Achieving World-Class Cancer Outcomes: A Strategy for England 2015–2020. London, 2015.

[2] Cancer Australia Cancer Australia Strategic Plan 2014–2019. Surrey Hills, 2014.

[3] Stubbings S, Robb K, Waller J et al Development of a measurement tool to assess public awareness of cancer. Br J Cancer 2009;101(Suppl):S13–7. 19956157 10.1038/sj.bjc.6605385 PMC2790699

[4] Robb K, Stubbings S, Ramirez AJ et al Public awareness of cancer in Britain: a population-based survey of adults. Br J Cancer 2009;101:S18–23. 10.1038/sj.bjc.6605386 PMC2790705 19956158

[5] Niksic M, Rachet B, Duffy SW et al Is cancer survival associated with cancer symptom awareness and barriers to seeking medical help in England? An ecological study. Br J Cancer 2016;7:876–86. 10.1038/bjc.2016.246 PMC5046204 27537388

[6] Waller J, Robb K, Stubbings S et al Awareness of cancer symptoms and anticipated help seeking among ethnic minority groups in England. Br J Cancer 2009;101:S24–30. 19956159 10.1038/sj.bjc.6605387 PMC2790709

[7] Moffat J, Bentley A, Ironmonger L et al The impact of national cancer awareness campaigns for bowel and lung cancer symptoms on sociodemographic inequalities in immediate key symptom awareness and GP attendances. Br J Cancer 2015;112:S14–21. 25734383 10.1038/bjc.2015.31 PMC4385971

[8] Power E, Wardle J Change in public awareness of symptoms and perceived barriers to seeing a doctor following Be Clear on Cancer campaigns in England. Br J Cancer 2015;112(Suppl):S22–6. 25734386 10.1038/bjc.2015.32 PMC4385972

[9] Public Health England *Be Clear on Cancer* 2016 http://www.nhs.uk/be-clear-on-cancer/.

[10] Public Health England *Be Clear on Cancer—Current Campaigns* 2016 http://www.cancerresearchuk.org/health-professional/early-diagnosis-activities/be-clear-on-cancer.

[11] NHS Scotland *Get Checked Early* 2012 http://www.getcheckedearly.org/.

[12] Cancer Australia *Lung Cancer Awareness Month.* Campaign. Events. 2013 https://canceraustralia.gov.au/healthy-living/campaigns-events/lung-cancer-awareness-month.

[13] Public Health England *Be Clear on Cancer—Abdominal Symptoms Campaign* 2016 http://www.cancerresearchuk.org/health-professional/early-diagnosis-activities/be-clear-on-cancer/abdominal-symptoms-campaign.

[14] Scott SE, Walter FM, Webster A et al The model of pathways to treatment: conceptualization and integration with existing theory. Br J Health Psychol 2013;18:45–65. 22536840 10.1111/j.2044-8287.2012.02077.x

[15] Lyratzopoulos G Markers and measures of timeliness of cancer diagnosis after symptom onset: a conceptual framework and its implications. Cancer Epidemiol 2014;38:211–3. 24742794 10.1016/j.canep.2014.03.009

[16] Ironmonger L, Ohuma E, Ormiston-Smith N et al An evaluation of the impact of large-scale interventions to raise public awareness of a lung cancer symptom. Br J Cancer 2014;112:207–16. 25461805 10.1038/bjc.2014.596 PMC4453621

[17] Koo MM, Hamilton W, Walter FM et al Symptom signatures and diagnostic timeliness in cancer patients: a review of current evidence. Neoplasia 2017;20(2):165–74. 29253839 10.1016/j.neo.2017.11.005 PMC5735300

[18] Rubin GP, McPhail S, Elliot K et al, Royal College of General Practitioners, Royal College of GPs *National Audit of Cancer Diagnosis in Primary Care* London, 2011 http://www.rcgp.org.uk/policy/rcgp-policy-areas/national-audit-of-cancer-diagnosis-in-primary-care.aspx.

[19] Lyratzopoulos G, Abel GA, McPhail S et al Gender inequalities in the promptness of diagnosis of bladder and renal cancer after symptomatic presentation: evidence from secondary analysis of an English primary care audit survey. BMJ Open 2013;3:e002861. 10.1136/bmjopen-2013-002861 PMC3693425 23798742

[20] Koo MM, von Wagner C, Abel G et al Typical and atypical symptoms in women with breast cancer: evidence of variation in diagnostic intervals from a national audit of cancer diagnosis. Cancer Epidemiol 2017;48:140–6. 28549339 10.1016/j.canep.2017.04.010 PMC5482318

[21] Nadkarni PM, Ohno-Machado L, Chapman WW Natural language processing: an introduction. J Am Med Inform Assoc 2011;18:544–51. 21846786 10.1136/amiajnl-2011-000464 PMC3168328

[22] NICE Suspected cancer: recognition and referral. 2015.

[23] Weller D, Vedsted P, Rubin G et al The Aarhus statement: improving design and reporting of studies on early cancer diagnosis. Br J Cancer 2012;106:1262–7. 22415239 10.1038/bjc.2012.68 PMC3314787

[24] Lyratzopoulos G, Neal RD, Barbiere JM et al Variation in number of general practitioner consultations before hospital referral for cancer: findings from the 2010 National Cancer Patient Experience Survey in England. Lancet Oncol 2012;13:353–65. 22365494 10.1016/S1470-2045(12)70041-4

[25] Ebell MH, Culp MB, Radke TJ A systematic review of symptoms for the diagnosis of ovarian cancer. Am J Prev Med 2016;50:384–94. 26541098 10.1016/j.amepre.2015.09.023

[26] Walter FM, Emery JD, Mendonca S et al Symptoms and patient factors associated with longer time to diagnosis for colorectal cancer: results from a prospective cohort study. Br J Cancer 2016;115:533–41. 27490803 10.1038/bjc.2016.221 PMC4997546

[27] Walter FM, Mills K, Mendonça SC et al Symptoms and patient factors associated with diagnostic intervals for pancreatic cancer (SYMPTOM pancreatic study): a prospective cohort study. Lancet Gastroenterol Hepatol 2016;1:298–306. 28404200 10.1016/S2468-1253(16)30079-6 PMC6358142

[28] Lyratzopoulos G, Saunders CL, Abel GA et al The relative length of the patient and the primary care interval in patients with 28 common and rarer cancers. Br J Cancer 2015;112:S35–40. 25734380 10.1038/bjc.2015.40 PMC4385974

[29] Keeble S, Abel GA, Saunders CL et al Variation in promptness of presentation among 10,297 patients subsequently diagnosed with one of 18 cancers: evidence from a National Audit of Cancer Diagnosis in Primary Care. Int J Cancer 2014;135:1220–8. 24515930 10.1002/ijc.28763 PMC4277322

[30] Stapley S, Peters TJ, Neal RD et al The risk of oesophago-gastric cancer in symptomatic patients in primary care: a large case-control study using electronic records. Br J Cancer 2013;108:25–31. 23257895 10.1038/bjc.2012.551 PMC3553533

[31] Elnegaard S, Andersen RS, Pedersen AF et al Self-reported symptoms and healthcare seeking in the general population—exploring ‘The Symptom Iceberg’. BMC Public Health 2015;15:685. 26195232 10.1186/s12889-015-2034-5 PMC4509464

[32] Hamilton W, Round A, Sharp D et al Clinical features of colorectal cancer before diagnosis: a population-based case-control study. Br J Cancer 2005;93:399–405. 16106247 10.1038/sj.bjc.6602714 PMC2361578

[33] Cancer Research UK *Be Clear on Cancer Programme Evaluation* Early diagnosis Act. 2016 http://www.cancerresearchuk.org/health-professional/early-diagnosis-activities/be-clear-on-cancer/programme-evaluation.

[34] Ewing M, Naredi P, Nemes S et al Increased consultation frequency in primary care, a risk marker for cancer: a case–control study. Scand J Prim Health Care 2016;34:205–12. 27189513 10.1080/02813432.2016.1183692 PMC4977944

[35] Abel GA, Saunders CL, Lyratzopoulos G Post-sampling mortality and non-response patterns in the English Cancer Patient Experience Survey: implications for epidemiological studies based on surveys of cancer patients. Cancer Epidemiol 2016;41:34–41. 26797675 10.1016/j.canep.2015.12.010 PMC4819677

[36] Hansen RP, Vedsted P, Sokolowski I et al Time intervals from first symptom to treatment of cancer: a cohort study of 2,212 newly diagnosed cancer patients. BMC Health Serv Res 2011;11:284. 22027084 10.1186/1472-6963-11-284 PMC3217887

[37] Leiva A, Esteva M, Llobera J et al Time to diagnosis and stage of symptomatic colorectal cancer determined by three different sources of information: a population based retrospective study. Cancer Epidemiol 2017;47:48–55. 28126583 10.1016/j.canep.2016.10.021

[38] Stapley SA, Rubin GP, Alsina D et al Clinical features of bowel disease in patients aged <50 years in primary care: a large case-control study. Br J Gen Pract 2017;67:e336–44. 28347985 10.3399/bjgp17X690425 PMC5409433

[39] Whitaker KL, Scott SE, Winstanley K et al Attributions of cancer ‘alarm’ symptoms in a community sample. PLoS One 2014;9:e114028. 25461959 10.1371/journal.pone.0114028 PMC4252079

[40] Elnegaard S, Pedersen AF, Andersen RS et al What triggers healthcare-seeking behaviour when experiencing a symptom? Results from a population-based survey. BJGP Open 2017;1:BJGP-2016-0775. 10.3399/bjgpopen17X100761 PMC6169954 30564656

[41] Macleod U, Mitchell ED, Burgess C et al Risk factors for delayed presentation and referral of symptomatic cancer: evidence for common cancers. Br J Cancer 2009;101(Suppl):S92–101. 19956172 10.1038/sj.bjc.6605398 PMC2790698

[42] McCutchan GM, Wood F, Edwards A et al Influences of cancer symptom knowledge, beliefs and barriers on cancer symptom presentation in relation to socioeconomic deprivation: a systematic review. BMC Cancer 2015;15:1000. 26698112 10.1186/s12885-015-1972-8 PMC4688960

[43] Salika T, Lyratzopoulos G, Whitaker KL et al Do comorbidities influence help-seeking for cancer alarm symptoms? A population-based survey in England. J Public Health (Oxf) 2017:1–10. https://academic.oup.com/jpubhealth/advance-article/doi/10.1093/pubmed/fdx072/3887249. 10.1093/pubmed/fdx072 PMC6105929 28655212

