# Supplementary material for: The nature and frequency of abdominal symptoms in cancer patients and their associations with time to help-seeking: evidence from a national audit of cancer diagnosis
Source: J Public Health (Oxf). 2018 Jan 27;40(3):e388–95. doi: 10.1093/pubmed/fdx188 (PMC6166582; doi:10.1093/pubmed/fdx188)
Supplement: Supplementary Data [file fdx188_jph_supplementary_18.12.17.docx]

# Supplemental tables and figures

**Figure S1** Flow chart describing sample derivation

**Table S2** Abdominal symptom definitions

**Table S3** Summary statistics for the patient interval (measured in days) and proportion of patients that experienced intervals exceeding 60 days, by abdominal symptom

**Table S4** Multivariate GLM output

**Table S5** Summary statistics for the patient interval (measured in days) and proportion of patients that experienced intervals exceeding 60 days, by abdominal symptom construct

**Table S6** Frequency of 12 most common abdominal symptom combinations

**Figure S7** Summary statistics for the patient interval (measured in days) and proportion of patients that experienced intervals exceeding 60 days, by symptom combination

## Figure S1 Flow chart describing sample derivation

Excluded 357 (2.2%)

Patients missing age or sex

16,374 patients

16,949 symptomatic patients with one of 29 cancer diagnoses* in the NACDPC 2009/10

Excluded 575 (3.4%)

Patients with an invalid symptom

15,956 patients with valid symptom information

Excluded 61 (0.4%)

Patients younger than 15 years

16,017 patients

*includes patients with cancer site categorised as ‘Other’

## Table S2 Abdominal symptom definitions

| **Symptom** | **Symptom constructs** |
| --- | --- |
|  |  |
| Abdominal Pain | Abdominal pain^1^ |
|  | Epigastric pain |
|  | Right Iliac Fossa (RIF) pain |
|  | Suprapubic pain |
|  | Loin pain & renal colic |
| Change in Bowel Habit | Constipation |
|  | Change in bowel habit |
|  | Diarrhoea |
| Dyspepsia | Dyspepsia and related epigastric symptoms^2^ |
| Dysphagia | Odynophagia |
|  | Dysphagia |
| Reflux | Reflux |
| Bloating or Distension | Abdominal bloating |
|  | Abdominal distension |
|  | Ascites |
| Nausea or Vomiting | Vomiting |
|  | Nausea |
| Rectal bleeding | Per-rectal bleeding^3^ |

^1^ Abdominal pain that was not otherwise specified, excluding acute abdominal pain

^2^ includes dyspepsia, indigestion, waterbrash, gastritis, burping, belching, “GI upset”, and “upper GI symptoms”.

^3^ includes blood in stool and rectal bleeding, excludes acute per rectal (PR) bleed

## Table S3 Summary statistics for the patient interval (measured in days) and proportion of patients that experienced intervals exceeding 60 days, by abdominal symptom

| **Symptom** | **N** | **Mean** | **25th** | **50th^a^** | **75th** | **90th** | **% 60+ days** |
| --- | --- | --- | --- | --- | --- | --- | --- |
| Abdominal pain | 670 | 28 | 0 | 7 | 28 | 70 | 12% |
| Change in bowel habit | 525 | 63 | 4 | 30 | 73 | 182 | 32% |
| Rectal bleeding | 495 | 55 | 1 | 16 | 59 | 136 | 25% |
| Dysphagia | 267 | 48 | 10 | 30 | 61 | 116 | 25% |
| Dyspepsia | 118 | 31 | 0 | 14 | 30 | 87 | 15% |
| Bloating or distension | 96 | 48 | 0 | 27 | 62 | 118 | 30% |
| Nausea or vomiting | 53 | 49 | 0 | 7 | 23 | 183 | 21% |
| Reflux | 29 | 41 | 0 | 16 | 61 | 128 | 27% |
| All abdominal symptoms | 2253 | 47 | 1 | 16 | 54 | 122 | 23% |

^a^ Kruskal-Wallis test of median values: p<0.001

NB 21% of all observations had missing information on the patient interval. For exact proportion by symptom category please see Table S5.

## Table S4 Multivariate GLM output

|  | **Patient interval** | | |
| --- | --- | --- | --- |
|  | **Coefficient** | **P value** | **Joint Wald test P value (overall)** |
| Abdominal symptom | . |  |  |
| Abdominal pain | (ref) | - | **<0.001** |
| Change in bowel habit | 2.5 (1.9–3.4) | **<0.001** |  |
| Rectal bleeding | 2.2 (1.5–3.1) | **<0.001** |  |
| Dysphagia | 1.7 (1.3–2.4) | **0.001** |  |
| Dyspepsia | 1.1 (0.6–2.0) | 0.766 |  |
| Bloating or distension | 2.1 (1.3–3.2) | 0.002 |  |
| Nausea or vomiting | 2.1 (0.6–6.7) | 0.235 |  |
| Reflux | 1.4 (0.7–2.9) | 0.373 |  |
| Sex |  |  |  |
| Male | (ref) | - | **0.013** |
| Female | 0.8 (0.6–1.0) | 0.013 |  |
| Age group |  |  |  |
| 15-49 years | 1.3 (0.9–1.9) | 0.193 | 0.209 |
| 60-69 years | (ref) | - |  |
| 70+ years | 0.8 (0.7–1.0) | 0.334 |  |
| Ethnicity |  |  |  |
| White | (ref) | - | 0.281 |
| Non-white | 1.3 (0.8–2.3) | 0.281 |  |

Bold indicates p<0.05.

NB n=1559 due to missing observations of ethnicity and patient interval. Specifically, 12% of patients with a single abdominal symptom had missing ethnicity while 21% had missing information on the patient interval. For exact proportions of missingness by symptom category please see Table S5.

## Table S5 Summary statistics for the patient interval (measured in days) and the proportion of patients that experienced intervals exceeding 60 days, by abdominal symptom construct

NB Variation in the patient interval was studied among those with a single symptom only, and so symptom constructs are mutually exclusive.

| **Symptom construct** | **Symptom** | **N** | **% (95% CI)** | **Mean** | **25th** | **50th** | **75th** | **90th** | **% 60+ days** | **% missing** |
| --- | --- | --- | --- | --- | --- | --- | --- | --- | --- | --- |
| Abdominal Pain | Abdominal pain^1^ | 515 | 23% (21%-25%) | 27 | 0 | 7 | 28 | 62 | 12% | 26% |
|  | Epigastric pain | 86 | 4% (3%-5%) | 39 | 3 | 12 | 31 | 105 | 17% | 26% |
|  | Loin pain & renal colic | 47 | 2% (2%-3%) | 22 | 0 | 2 | 31 | 61 | 13% | 17% |
|  | Right iliac fossa pain | 18 | 0.8% (0.5%-1.3%) | 11 | 0 | 1 | 5 | 31 | 7% | 17% |
|  | Suprapubic pain | 4 | 0.2% (0.1%-0.5%) | 0 | 0 | 0 | 0 | 0 | 0% | 25% |
| Change in Bowel Habit | Change in bowel habit | 262 | 12% (10%-13%) | 78 | 13 | 41 | 94 | 201 | 42% | 16% |
|  | Diarrhoea | 176 | 8% (7%-9%) | 57 | 3 | 27 | 61 | 151 | 26% | 15% |
|  | Constipation | 87 | 4% (3%-5%) | 29 | 1 | 7 | 27 | 83 | 15% | 25% |
| Dyspepsia | Dyspepsia and related epigastric symptoms^2^ | 118 | 5% (4%-6%) | 31 | 0 | 14 | 30 | 87 | 15% | 25% |
| Dysphagia | Dysphagia | 258 | 11% (10%-13%) | 49 | 10 | 30 | 61 | 118 | 26% | 16% |
|  | Odynophagia | 9 | 0.4% (0.2%-0.8%) | 17 | 0 | 13 | 31 | 60 | 0% | 22% |
| Reflux | Reflux | 29 | 1.3% (0.9%-1.8%) | 41 | 0 | 16 | 61 | 128 | 27% | 24% |
| Bloating or Distension | Abdominal bloating | 60 | 3% (2%-3%) | 46 | 3 | 26 | 62 | 92 | 27% | 27% |
|  | Abdominal distension | 27 | 1.2% (0.8%-1.7%) | 50 | 0 | 28 | 92 | 123 | 36% | 19% |
|  | Ascites | 9 | 0.4% (0.2%-0.8%) | 61 | 0 | 16 | 122 | 213 | 25% | 56% |
| Nausea or Vomiting | Vomiting | 35 | 2% (1%-2%) | 24 | 1 | 4 | 17 | 105 | 12% | 51% |
|  | Nausea | 18 | 0.8% (0.5%-1.3%) | 85 | 0 | 15 | 98 | 183 | 33% | 33% |
| Rectal bleeding | Per-rectal bleeding^3^ | 495 | 22% (20%-24%) | 55 | 1 | 16 | 59 | 136 | 25% | 16% |
| All abdominal symptoms | - | 2253 | 100% | 47 | 1 | 16 | 54 | 122 | 23% | 21% |

^1^ Abdominal pain that was not otherwise specified, excluding acute abdominal pain

^2^ includes dyspepsia, indigestion, waterbrash, gastritis, burping, belching, “GI upset”, and “upper GI symptoms”.

^3^ includes blood in stool or rectal bleeding, excludes acute per rectal (PR) bleed

## Table S6 Frequency of 12 most common abdominal symptom combinations

|  | **Frequency of 12 most common abdominal symptom combinations among symptomatic cancer patients (n=15,956)** | |
| --- | --- | --- |
| **Symptom combination*** | **No. of patients** | **% (95% CI)** |
| Abdominal pain alone | 952 | 6.0% (5.6% – 6.3%) |
| CIBH alone | 694 | 4.3% (4.0% - 4.7%) |
| Rectal bleeding alone | 595 | 3.7% (3.4% - 4.0%) |
| Dysphagia alone | 355 | 2.2% (2.0% - 2.5%) |
| Dyspepsia alone | 168 | 1.1% (0.9% – 1.2%) |
| **CIBH & Rectal bleeding** | 147 | 0.9% (0.8% – 1.1%) |
| Nausea or vomiting alone | 140 | 0.9% (0.7% – 1.0%) |
| Bloating or distension alone | 138 | 0.9% (0.7% – 1.0%) |
| **Abdominal pain & CIBH** | 93 | 0.6% (0.5% – 0.7%) |
| **Abdominal pain & Nausea or vomiting** | 64 | 0.4% (0.3% – 0.5%) |
| **Abdominal pain & Bloating or distension** | 56 | 0.4% (0.3% – 0.5%) |
| Reflux alone | 36 | 0.2% (0.2% – 0.3%) |

CIBH = change in bowel habit. Symptom pairs are in bold print.

*Symptom combinations based on presence/absence of abdominal symptoms only.

Patients with the 12 most frequent symptom combinations were selected for supplementary analyses (n=3,438, 94% of all patients with an abdominal symptom in our sample). This included the eight abdominal symptoms as single symptoms, as well as four symptom pairs: change in bowel habit (CIBH) and rectal bleeding; abdominal pain and CIBH; abdominal pain and nausea/vomiting; and abdominal pain and bloating/distension (see table S6).

Change in bowel habit and rectal bleeding was the most common symptom pair (seen in 0.9% of all symptomatic cancer patients).

## Table S7 Summary statistics for the patient interval (measured in days) and proportion of patients that experienced intervals exceeding 60 days, by symptom combination

| **Symptom** | **N** | **Mean** | **25th** | **50th ^a^** | **75th** | **90th** | **% 60+ days** |
| --- | --- | --- | --- | --- | --- | --- | --- |
| Abdominal pain | 952 | 29 | 0 | 7 | 30 | 77 | 14% |
| CIBH | 694 | 62 | 4 | 30 | 73 | 178 | 32% |
| Rectal bleeding | 595 | 56 | 1 | 17 | 61 | 136 | 26% |
| Dysphagia | 355 | 49 | 9 | 30 | 61 | 118 | 25% |
| Dyspepsia | 168 | 41 | 0 | 17 | 38 | 92 | 19% |
| **CIBH & Rectal bleeding** | 147 | 59 | 8 | 33 | 90 | 127 | 38% |
| Nausea or vomiting | 140 | 43 | 2 | 14 | 33 | 99 | 17% |
| Bloating or distension | 138 | 41 | 2 | 17 | 58 | 109 | 25% |
| **Abdominal pain & CIBH** | 93 | 44 | 6 | 18 | 49 | 124 | 23% |
| **Abdominal pain & Nausea or vomiting** | 64 | 24 | 1 | 14 | 35 | 67 | 14% |
| **Abdominal pain & bloating or distension** | 56 | 44 | 1 | 19 | 58 | 143 | 23% |
| Reflux | 36 | 36 | 3 | 11 | 57 | 128 | 21% |

CIBH = change in bowel habit. Symptom pairs are in bold print.

NB 20% of all observations (n=3438) had missing information on the patient interval.

*Not calculable as median primary care interval value was zero

^a^ Kruskal-Wallis test of median values; p<0.001

The variation observed in the patient interval by abdominal symptom combination across the 12 examined groups was similar to that of individual symptoms (see Table 3 in main text and Table S7).

Patients with abdominal pain alone had the shortest median time to presentation (median (IQR): 7 (0–30) days). Patients who had one of three symptom pairs including abdominal pain waited longer before seeking help (median patient interval: 14–19 days). Patients who presented with CIBH and rectal bleeding had a longer patient interval compared to those who had change in bowel habit or rectal bleeding alone (median patient interval: 33 days vs. 30 days and 17 days respectively).
